# An evaluation of the prevalence of weight-related comorbidities in patients following admission to the general adult inpatient wards in Mersey Care NHS Foundation Trust

**DOI:** 10.1192/bjo.2021.259

**Published:** 2021-06-18

**Authors:** Declan Hyland, Millie Prime, Rabia Khaliq

**Affiliations:** 1 Consultant Psychiatrist, Clock View Hospital, Liverpool, Mersey Care NHS Foundation Trust; 2 5th year medical undergraduate, University of Liverpool

## Abstract

**Aims:**

This audit aims to evaluate the prevalence of any weight-related comorbidities in patients following their admission to the general adult inpatient wards in Mersey Care NHS Foundation Trust.

**Background:**

Obesity has an increased prevalence in those with mental disorder. There are many factors that influence this, e.g. sedentary lifestyle and poor dietary intake. Medication prescribed to treat mental disorders may increase risk of weight gain, particularly most of the second generation antipsychotics. Patients with severe mental illness are at increased likelihood of developing weight-related comorbidities - essential hypertension, ischaemic heart disease, hyperlipidaemia and type II diabetes mellitus.

Many patients with severe and enduring mental illness do not readily or regularly access primary care services. Admission to the psychiatric ward therefore provides an ideal opportunity to address, not only the patient's mental health issues, but also any physical health issues.

**Method:**

A list of all inpatients on the eight general adult wards was obtained on 3rd of December 2020. Inpatients on the Psychiatric Intensive Care Unit were also incorporated, providing a final sample of 148 inpatients.

An audit tool was designed, to collect demographic data for each inpatient – gender, age, ethnicity, psychiatric diagnosis, as well as the presence of any cardiovascular comorbidities and, if so, what were they and how many were present.

**Result:**

Of the 148 inpatients, 91 were male, 57 female. Patient age ranged from 19 to 71 years. The majority were of “white British” ethnicity. The most common mental disorder diagnosis was schizophrenia (35 inpatients), followed by schizoaffective disorder (22 inpatients). Twenty-one of the 148 patients had at least one weight-related comorbidity recorded. Only 2 of the 21 inpatients with a diagnosis of one or more weight-related comorbidity had a recorded BMI in the “healthy” range. The gender split for the presence of weight-related comorbidities was almost equal. The most common comorbidity recorded was type II diabetes mellitus. Most patients with a weight-related comorbidity had only one recorded, but three patients had two comorbidities recorded, and one patient had three recorded.

**Conclusion:**

A significant proportion of patients admitted to the general adult inpatient wards in the trust have a weight-related comorbidity. Admission to hospital provides an ideal opportunity to review the management of any such comorbidity and optimise this as required. There is a need to ensure there is a strong focus on, not only the patient's mental health issues, but also his / her physical health status.

